# Novel NIR fluorescent probe IR-546 inhibits melanoma through the AKT/GSK3β/β-catenin pathway

**DOI:** 10.1186/s10020-025-01289-0

**Published:** 2025-06-10

**Authors:** Hongye Liao, Tong Xia, Ziyuan Zeng, Xun Yang, Simei Yang, Xia Xiong, Yuanmin He, Changzhen Sun, Na Hao, Li Liu

**Affiliations:** 1 Department of Dermatology, The Affiliated Hospital, Southwest Medical University, Luzhou, 646000 China; 2 School of Public Health, Southwest Medical University, Luzhou, 646000 China; 3 Drug Research Center of Integrated Traditional Chinese and Western Medicine, The Affiliated Traditional Chinese Medicine Hospital, Southwest Medical University, Luzhou, 646000 China; 4 Department of Medicinal Chemistry, Green Pharmaceutical Technology Key Laboratory of Luzhou, Central Nervous System Drug Key Laboratory of Sichuan Province, School of Pharmacy, Southwest Medical University, Luzhou, 646000 China

**Keywords:** Near-infrared (NIR) fluorescent probe, Malignant melanoma, Apoptosis, Cyanine dyes, Mitochondrial-targeted

## Abstract

**Background:**

Melanoma is a highly invasive and metastatic skin cancer lacking effective options for early diagnosis and treatment. To address these challenges, this study aimed to synthesize a novel near-infrared fluorescent probe and research the antitumor mechanism of melanoma.

**Methods:**

We have designed and synthesized a novel near-infrared fluorescent probe, IR-546. This study explores imaging capabilities, anti-tumor effects, and underlying mechanisms of IR-546 in melanoma xenograft models.

**Results:**

In vitro, IR-546 selectively accumulates in melanoma cells, demonstrating robust tumor imaging and potent cytotoxicity by targeting mitochondria, generating reactive oxygen species (ROS), and disrupting mitochondrial membrane potential, activating the mitochondrial apoptotic pathway. Additionally, IR-546 exhibits anti-metastatic properties in vitro. In vivo, using A375 cell line-derived melanoma xenograft models, IR-546 showed significant anti-tumor effect and biosafety. Western blot analyses of both in vivo and in vitro revealed that IR-546 induces apoptosis and inhibits metastasis of melanoma by activating the mitochondrial apoptosis pathway, suppressing the AKT/GSK3β signaling pathway, and downregulating the β-catenin signaling pathway and its downstream targets. Immunohistochemistry and immunofluorescence further confirmed that IR-546 suppressed the expression of key proteins in the AKT/GSK3β pathway in vivo.

**Conclusions:**

Collectively, these findings highlight IR-546 as a promising tool for both imaging and treatment of melanoma, with the potential to induce apoptosis and inhibit metastasis of melanoma through modulation of the AKT/GSK3β pathway.

**Graphical Abstract:**

Summary of the diagnostic and anti-tumor effects of IR-546 in melanoma.

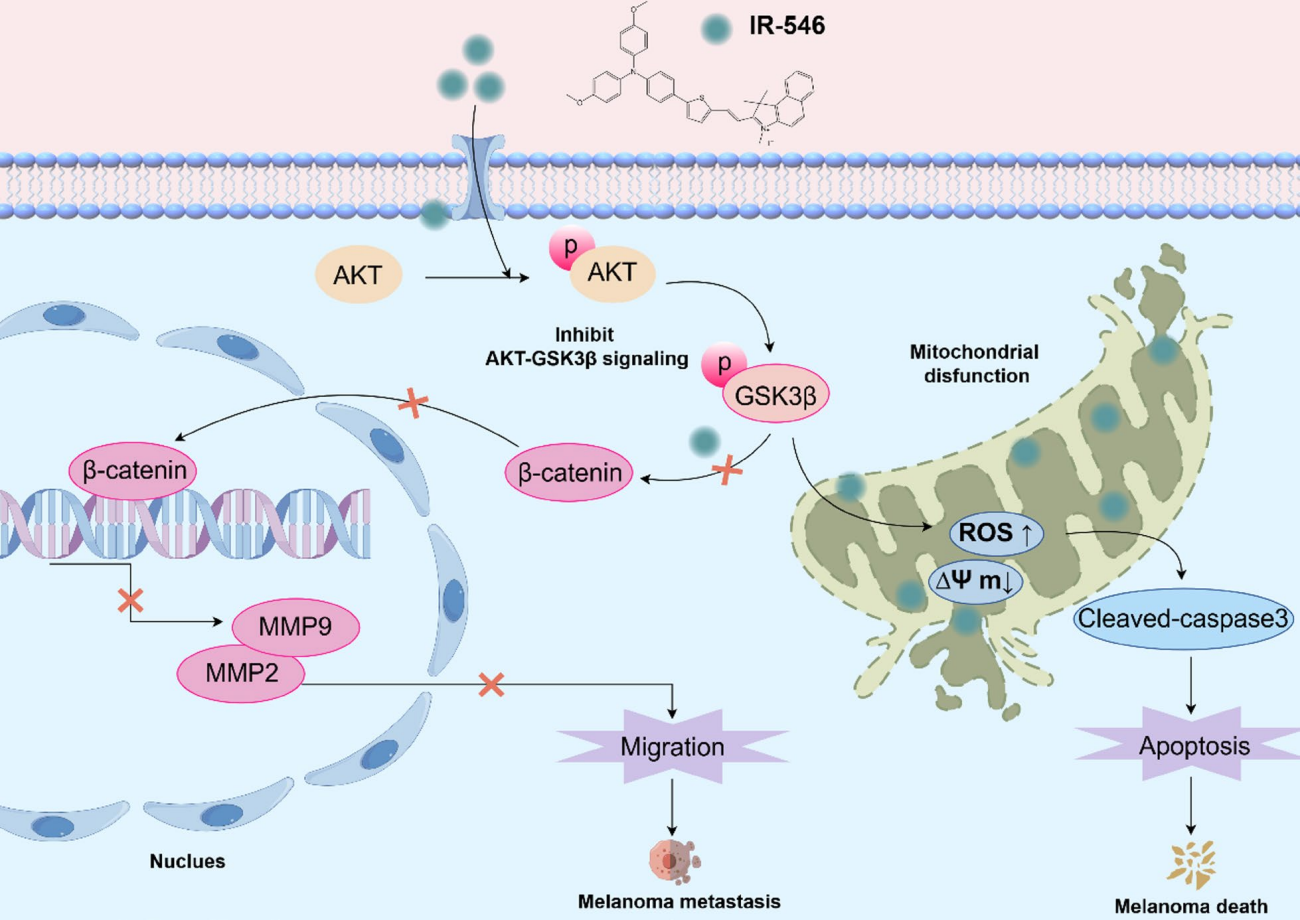

**Supplementary Information:**

The online version contains supplementary material available at 10.1186/s10020-025-01289-0.

## Introduction

Melanoma is a highly aggressive and metastatic skin cancer, with incidence rates continuing to rise worldwide (Bray et al., 2018). By 2040, new melanoma cases and deaths are projected to increase by 56% and 68%, respectively (Arnold et al., 2022). Early-stage melanoma can be treated surgically, but once metastasis occurs, survival rates drop significantly (Walz et al., 2022). For instance. the 5-year survival rate for primary melanoma is 99%, but for metastatic melanoma, it falls to just 27% (Siegel et al., 2021). Late diagnoses, often associated with distant metastases result in missed treatment opportunities (Gray-Schopfer et al., 2007), making early detection and intervention critical to improving patient survival.

Mitochondria, the central energy metabolism hub, play a crucial role in tumor growth and apoptosis (Weinberg and Chandel, 2015). Thus, mitochondria have become a target for cancer therapies (Borcherding and Brestoff, 2023, Kadkhoda et al., 2022). Clinical trials have shown that disrupting mitochondrial functions – such as the electron transport chain (ETC), nucleotide metabolism, and the Krebs cycle – can induce apoptosis in tumor cells (Vasan et al., 2020, Sainero-Alcolado et al., 2022). Mitochondria are also involved in other key cellular processes, such as generating reactive oxygen species (ROS), and calcium homeostasis (Rodrigues and Ferraz, 2020), with ROS playing a pivotal role in melanoma progression (Liu-Smith et al., 2014). ROS production includes chemically reactive molecules such as superoxide anions, peroxides, and hydroxyl radicals (Ziech et al., 2011). Excessive ROS production can induce oxidative stress, promoting signaling pathways that inhibit tumor survival (Chan et al., 2009). Given the negative membrane potential and unique lipid bilayer of mitochondria, drugs with cationic and lipophilic properties are particularly effective in targeting them (Wang et al., 2017). Near-infrared (NIR) fluorescent probes, such as cyanine-based compounds, have been extensively studied for tumor diagnosis and therapy (St Lorenz et al., 2021). Tumor cells exhibit greater mitochondrial membrane potential polarization than normal cells, facilitating selective drug uptake (Cheung and Vousden, 2022). Hence, developing novel mitochondrial-targeted probes offers substantial potential for melanoma diagnosis and treatment.

NIR fluorescent probes are gaining traction due to their tissue penetration and tumor-targeting properties (Li et al., 2022b). Modifying their functional groups can enhance spectral characteristics, targeting specificity, water solubility, and biocompatibility (Guo et al., 2021; Li et al., 2022a; Dai et al., 2021). For example, the NIR probe 808-NM2, a modified version of IR-808, has demonstrated mitochondrial targeting and therapeutic efficacy in breast cancer imaging and chemotherapy (Chen et al., 2021). Similarly, our previous work with the NIR probe, IR-817, showed its effectiveness in targeting melanoma cell mitochondria for both diagnosis and treatment (Sun et al., 2022; Wang et al., 2023b).

The Wnt/β-catenin pathway is crucial for tumor invasion (Zhang and Wang 2020; Zhao et al., 2022). Glycogen synthase kinase-3β (GSK3β), an evolutionarily conserved serine-threonine protein kinase, plays a key role in regulating this pathway (Aghabozorgi et al., 2020). GSK3β activity is modulated by its phosphorylation: Ser9 phosphorylation inhibits, while Tyr216 phosphorylation activates it. Phosphorylation of β-catenin at Ser33/37/Thr47 by GSK3β prevents β-catenin from entering the nucleus, thereby inhibiting the expression of its downstream target genes (Dai et al., 2024). In addition to its role in the Wnt/β-catenin pathway, GSK3β is a multifunctional regulator of mitochondrial energy metabolism and apoptosis (Yang et al., 2017). Given these diverse roles, GSK3β is considered a promising target in cancer therapy (Albrecht et al., 2021). For instance, Xu et al. reported that mitochondrial-driven apoptosis in the retina was reduced by upregulating the AKT/GSK3β/β-catenin pathway (Xu et al., 2022). while Leost M et al. found that GSK3β inhibitors were used effectively in treating advanced prostate cancer (Leost et al., 2000). Recent studies have also focused on the AKT/GSK3β pathway in melanoma. Chen et al. showed that eEF2 K inactivates GSK3β by phosphorylating it at Ser9, thereby promoting tumor immune escape and melanoma growth (Chen et al., 2022). Similarly, Wang et al. demonstrated that SLFN5 suppresses melanoma cell migration and invasion by inhibiting MT1-MMP expression through AKT/GSK-3β/β-catenin signaling pathway (Wan et al., 2019). Prior studies have shown that Kaempferol (KAM) inhibits melanoma metastasis by impairing glycolysis through the AKT/GSK-3β pathway(Zheng et al., 2022). Similarly, SGK3 overexpression has been reported to induce GSK-3β phosphorylation, increasing breast cancer cell proliferation, decreasing apoptosis, and enhancing invasion and migration(Sun et al., 2016). These findings suggest that the AKT/GSK3β pathway plays a pivotal role in melanoma progression, warranting further investigation. In this study, we focused on GSK3β, a key regulator of tumor apoptosis and metastasis. We aim to explore the expression of proteins upstream and downstream of GSK3β, to understand better its role in inducing tumor apoptosis and inhibiting metastasis.

We synthesized a novel NIR fluorescent probe, IR-546, designed to target mitochondria and investigated its cellular entry mechanisms and mitochondrial targeting in A375 and B16-F10 melanoma cell lines. Additionally, we assessed the imaging, anti-tumor, and anti-metastatic effects of IR-546 in vitro. Using A375 melanoma cells, we evaluated the probe’s antitumor efficacy and biosafety in vivo. Importantly, we found that IR-546 enters melanoma cells via energy metabolism-related pathways, disrupts mitochondrial function, and induces apoptosis in tumor cells. Furthermore, we demonstrated that IR-546 induces apoptosis and inhibits metastasis of tumor cells by suppressing the AKT/GSK3β signaling pathway in vitro and in vivo. Thus, our novel NIR fluorescent probe IR-546, shows significant potential as a therapeutic tool for both diagnosing and treating melanoma.

## Material and method

### Synthesis and characterization of IR-546

IR-546 was synthesized according to the procedure in references (Xiao et al., 2023; He et al., 2024; Wang et al., 2023a). The product was characterized by HRMS, ^1^H NMR and ^13^C NMR.

The absorption and emission spectra of 10 µM IR-546 in methanol, PBS, 10% FBS, DMSO and 0.9% NaCl solutions were determined by UV and fluorescence spectrophotometers, respectively.

###  Cell culture

A375, B16-F10, HeLa, COS-1, MDA-MB-231 and A549 cells were sourced from ATCC (Manassas, Virginia, USA) and cultured in Dulbecco Modified Eagle Medium (DMEM) containing 10% fetal bovine serum and 1% penicillin/streptomycin solution. B16-F10 was cultured in RPMI 1640 medium containing 10% fetal calf serum and 1% penicillin/streptomycin solution. All cells are at 37 °C and the cell incubation box culture contains 5% CO_2_.

### Cell imaging

To observe the fluorescence effect of IR-546 in different cells, A375, B16-F10, HeLa and COS-1 cells were inoculated in 6-well plates, and 20 µM IR-546 was added and incubated for 2 h after the cells were attached to the wall. Fluorescence images were captured using a live cell workstation.

### Cell viability and live-dead staining

Cell viability was quantified in A375 and B16-F10 melanoma cells using an MTT assay and Calcein/PI Cell Viability/Cytotoxicity Assay Kit to assess the antiproliferative effect.

A375, B16-F10, COS-1, HeLa, MDA-MB-231 and A549 cells were seeded in 96 well plates at a density of 2–6 × 10^3^ cells per well respectively. Waiting for overnight adherence, cells were incubated with medium alone or with IR-546 (0, 0.63, 1.25, 2.5, 5 and 10 µM) at 37 °C for 24 h, 48 h and 72 h, respectively, and then cell proliferation was determined by MTT assay. Cells were treated with MTT solution (The final concentration of 0.5 mg/ml) for 2–4 h. The supernatant was discarded, followed by the addition of 150 µL DMSO to each well to dissolve the precipitate. Finally, the absorbance of the well plate at 570 nm was measured using a full-wavelength microplate reader.

Calcein combined with PI can simultaneously double the fluorescence staining of live and dead cells. A375 and B16-F10 melanoma cells were treated with 2.5 µM IR-546 for 24 h, respectively. The operation was carried out according to the instructions of the kit, and finally, photos were taken with an inverted fluorescence microscope. Measurements were made three times for each experiment.

### Wound healing assay

The A375 and B16-F10 melanoma cells were inoculated into 6-well plates at a density of 3–5 × 10^5^ cells per well, and cultured into about 90% fused cell monolayers, which were then scraped with sterile 10 µL pipet tips and washed three times with PBS. Cultures were incubated with 2 ml of drug-containing medium containing IR-546 (0, 0.63, 1.25, 2.5, 5 µM). At 0, 12 and 24 h with a microscope photographs.

### Transwell migration assay

Cells were first starved overnight and re-suspended in serum-free medium, then 200 µL of IR-546 (0, 0.63, 1.25, 2.5, 5 µM) -containing serum-free medium was added and seeded in the upper chamber at a density of 2 × 10^5^ cells per well. 500 µL of medium containing 20% FBS was added to the lower chamber. The medium in the upper chamber was removed, fixed with 4% paraformaldehyde for 10 min, washed six times with PBS, and the medial non-migrating cells were carefully wiped with a cotton swab. Finally, the cells were stained with 1% crystal violet, and washed six times with PBS, and the migrating cells were observed and photographed under a microscope.

### Colocalization of IR-546 to mitochondria and determination of (MMP, ΔΨm)

According to the manufacturer’s protocol, mitochondrial membrane potential (MMP, ΔΨm) was measured using the JC-1 Mitochondrial Membrane Potential Assay kit (Solarbio). First, the cells were incubated for 24 h with a drug-containing medium containing 1.25 and 2.5 µM IR-546 and A375 and B16-F10 melanoma cells, stained with JC-1 for 20 min at 37 °C, washed twice with JC-1 buffer solution 1x, and finally observed by fluorescence microscopy. *E*_*x*_=490/525 nm and *E*_*m*_*=*530/590 nm were set for JC-1 monomer/polymer detection, respectively.

### The cellular uptake mechanism of IR-546

To investigate the cellular uptake mechanism of IR-546. A375 and B16-F10 melanoma cells were pretreated 30 min earlier with inhibitors (4 °C, 250 µM BSP, 5 mg/ml CPZ, 10 µM EIPA, 40 ug/ml Genistein). Then the culture medium was removed and the cells were treated with IR-546 for 2 h. The cell uptake was observed by an inverted microscope fluorescence microscope and fluorescence quantitative analysis was performed.

### Western blot analysis

First, the protein was extracted using RIPA lysate. Protein concentration was measured using the BCA kit. Proteins were separated by SDS-PAGE, transferred to PVDF membranes, blocked with rapid blocking solution for 10 min at room temperature, and incubated with the first antibody overnight at 4 °C. The second antibody was incubated for 90 min at room temperature. Blots were acquired using a chemiluminescence imaging system and ECL Western blotting kit scanning. Antibodies used for analysis were: Cleaved caspase-3 (341034, zenbio), Bcl-2 (AF6285, beyotime), β-actin (250136, zenbio), MMP2 (10373-2-AP, Proteintetech), MMP9 (502095, zenbio), β-catenin (AF0069, beyotime), AKT (342529, zenbio), Phospho-AKT (Ser473) (4060 S, CST), Phospho-GSK-3β (Ser 9) (310021, zenbio).

### In vivo experiment

BALB/c mice (male, 4–6 weeks old) were purchased from Chengdu Yaokang Biotechnology Co. LTD. A subcutaneous tumor model was constructed in the right gluteal muscle injection of 200 µL A375 human malignant melanoma cells (5 × 10^6^ cells/mice). All animal experiments were approved by the Laboratory Animal Management Committee of the Affiliated Hospital of Southwest Medical University (Permit Number: 20240112-001). And by approved guidelines.

 A375 human malignant melanoma cells were selected and subcutaneous xenograft models were constructed in BALB/c mice. The experimental groups were divided into three groups (*n* = 6): normal group:10%FBS, low concentration group: 0.5 mg/kg IR-546 and high concentration group: 2 mg/kg IR-546, The volume of each drug injection is 200 µL, and the treatment is administered every other day. When the tumor size reached about 100 mm^3^, treated every other day for eight treatment cycles. The body weight of the mice was recorded, and the tumor volume was measured (formula V (mm^3^) = ½ab^2^). The mice were sacrificed after 8 cycles of treatment. Extraction of tumor in mice and organs, in the evaluation of the IR-546 in the body of tumor suppression function and biological safety.

### H&E staining

The internal organs and tumor tissues of nude mice in different experimental groups were collected, fully fixed with 4% paraformaldehyde, then dehydrated with ethanol gradient, removed with xylene, and embedded in paraffin. Cut into 4 μm thick slices, stained with eosin and hematoxylin, sealed with resin, and viewed with a microscope.

### Immunohistochemistry

Immunohistochemical staining was performed using specific antibodies, including Ki67, CC-3, p-GSK-3β (Ser9), p-AKT and MMP2.

### Statistical analysis

All the experimental data in this paper were processed with Graph-Pad Prism 9.5 software, ImageJ software and Figdraw (www.figdraw.com, accessed data: 27 September 2024). One-way ANOVA and Unpaired Student’s t-test were applied to the analysis between groups. Statistical analyses were expressed as mean ± SD. *P* < 0.05 was statistically significant.

## Result and discussion

### Synthesis and optical properties of IR-546

**Fig. 1:**
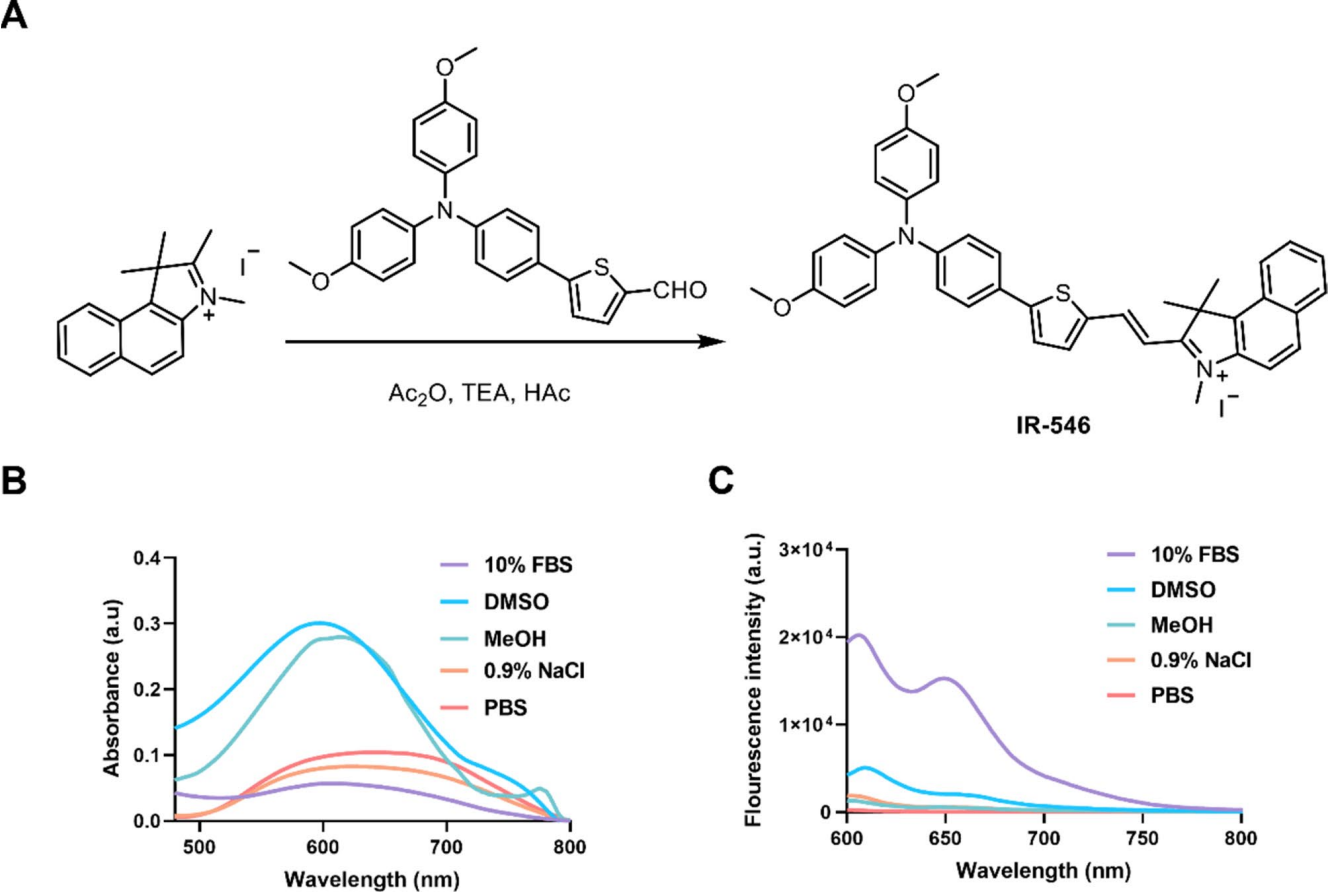
Synthesis and optical properties of IR-546. **A** The synthesis routes of IR-546. **B** Absorption spectrum of 10 µM IR-546 in MeOH, PBS, 10% FBS, DMSO, and 0.9% NaCl solvents. **C** Fluorescence spectrum of 10 µM IR-546 in MeOH, PBS, 10% FBS, DMSO, and 0.9% NaCl solvents

The synthesis route of IR-546 and its structural formula are shown in Fig. 1A. To synthesize IR-546, 1,2,3,3-Tetramethylbenz[e]in dolium iodide (98 mg, 0.28 mmol) and 5-(4-(bis(4-methoxyphenyl)amino)phenyl)thiophene-2-carbaldehyde(116 mg, 0.28 mmol)were dissolved in 0.2 mL acetic anhydride, followed by the addition of 0.2 mL triethylamine and 2 mL acetic acid. The reaction mixture was stirred at 60 ℃ for 1 h. After cooling to room temperature, 20 mL of diethyl ether (Et_2_O) was added, and the resulting solids were filtered and collected. The dark precipitate was further purified via column chromatography (silica gel) using a DCM/MeOH mixture (10:1 v/v) as eluent, yielding a black powder of IR-546. Yield: 92 mg, 43%. Cal. M^+^:C_41_H_37_ N_2_O2S^+^, 621.2570, found: 621.2570. The product was characterized by ^1^H NMR (400 MHz, DMSO-d_6_): δ = 1.99 (6 H, s), 3.77 (6 H, s), 4.16 (3 H, s), 5.76 (1H, s), 6.78–6.80 (2 H, d, J = 8.4), 6.97–6.99 (4 H, d, J = 8.8), 7.13–7.15 (4 H, d, J = 8.8), 7.64–7.72 (4 H, m), 7.77–7.81 (1H, dd, J = 8.4), 8.04–8.07 (1H, d, J = 9.2), 8.14–8.15 (1H, d, J = 4.0), 8.19–8.21 (1H, d, J = 8.0), 8.25–8.27 (1H, d, J = 8.8), 8.40–8.42 (1H, d, J = 8.4), 8.69–8.73 (1H, d, J = 15.6). 13 C NMR (DMSO-d6, 101 MHz): δ = 196.39, 157.07, 139.94, 139.33, 136.98, 133.50, 130.99, 130.22, 128.86, 128.15, 127.61, 123.89, 115.67, 113.62, 55.73, 46.24, 35.54, 25.93, 21.74, 14.43, 9.14 (Figs. S1- S3).

In the wavelength range of 600–900 nm exhibits excellent responsiveness to phototherapy (Vickerman et al., 2021). Fluorescent probes within this range can achieve targeted detection of mitochondria, lysosomes, and biomolecules by coupling different functional groups (Li et al., 2021). Therefore, NIR dyes in the range of 600 to 900 nm are considered the best choice for biological imaging due to their “tissue optical window” (Joshi et al., 2021; Shell et al., 2015). The optical properties of IR-546 were characterized through spectral analysis. Absorption and fluorescence spectra of IR-546 were recorded in various solvents, including methanol, PBS, 10% FBS, DMSO, and 0.9% NaCl (Fig. 1B, C). In all solvents, IR-546 exhibited an absorption peak at 600 nm, and an emission peak at 605 nm and 650 nm, confirming its near-infrared (NIR) fluorescence properties (600–900 nm). Notably, we observed the physical images of IR-546 in different solutions, which again confirmed that IR-546 had the best fluorescence signal in 10% FBS solution, and confirmed that IR-546 had good biological application potential in vitro (Fig. S4), the fluorescence intensity of IR-546 in 10% FBS was significantly higher than in other solvents, indicating its potential for in vivo bioimaging and therapeutic application.

### The selective targeting effect of IR-546 in vitro

**Fig. 2:**
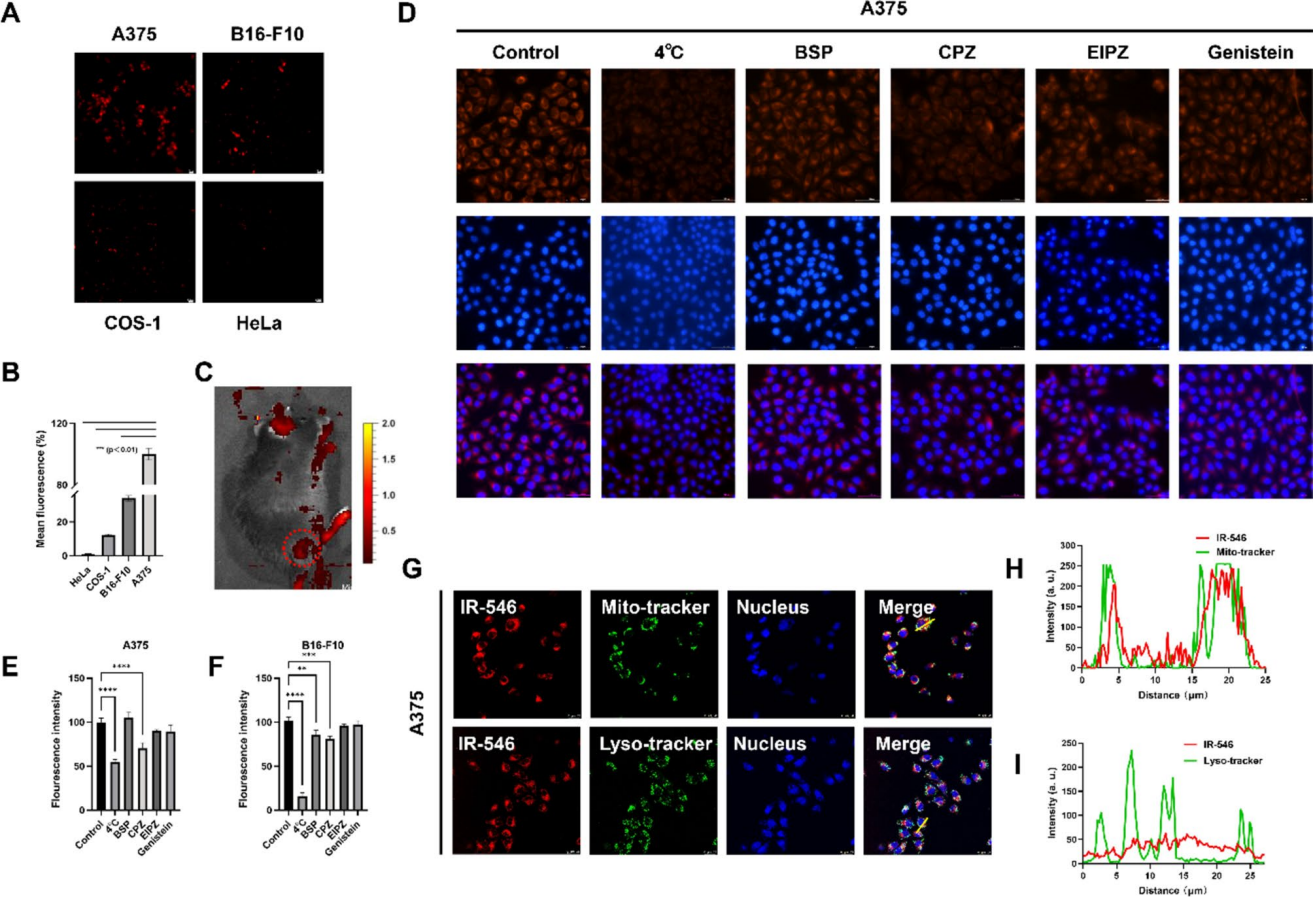
The selective targeting effect of IR-546 in vitro. **A** and **B** Fluorescence images of 20 µM IR-546 co-incubated with A375, B16-F10, HeLa, and COS-1 cells for 2 h, scale bar = 20 μm. **C** Fluorescence imaging of the mice at 2 h. **D** Fluorescence images of A375 melanoma cells after incubation with IR-546 and different endocytic inhibitors, scale bar = 100 μm. **E** and **F** Shows the A375 and B16-F10 melanoma cells and IR-546 within different inhibitors after incubation with fluorescent quantitative analysis. **G** Colocalization fluorescence imaging of IR-546 with Mito-Tracker and Lyso-Tracker in A375 melanoma cells, respectively, bar = 25 μm. **H** and **I** Show the colocalization analysis of IR-546 in mitochondria and lysosome in A375 melanoma cells, respectively. Each result was mean ± SD, *n* = 3. (****P < 0.0001, *** *P* < 0.001, ** *P* < 0.01, * *P* < 0.05, compared with control)

To evaluate the selective targeting of IR-546, we used fluorescence microscopy to observe its uptake in tumor and normal cells. IR-546 preferentially accumulated in tumor cells compared to normal cells, with melanoma cells, particularly A375 cells, showing a higher uptake within 2 h (Fig. 2A, B). We further investigated the imaging of IR-546 in vitro, we observed IR-546 was able to rapidly target melanoma cells in mice within 2 h (Fig. n 2C). These findings suggest that IR-546 has potential for melanoma imaging. To investigate the internalization pathway of IR-546, we pre-treated A375 and B16-F10 melanoma cells under different conditions, including lowering the incubation temperature to 4 °C (energy blockade), using sulfobromophthalein (BSP, competitive inhibitor of organic anion transporting polypeptides), chlorpromazine (CPZ, inhibitor of clathrin-dependent endocytosis), ethylisopropylamiloride (EIPA; inhibitor of macropinocytosis), and genistein (GEN, inhibitor of caveolin-dependent endocytosis). Quantitative analysis of intracellular fluorescence intensity showed that IR-546 uptake was significantly reduced in the 4 °C, BSP, and GEN groups (Fig. 2D, S5), with the lowest fluorescence observed in the 4 °C group. This indicates that IR-546 uptake in A375 and B16-F10 is mainly energy-dependent and partially mediated by OATP transporters and clathrin-dependent endocytosis (Fig. 2E, F). IR-546 is a NIR probe based on hemicyanine modification, and the cellular uptake pathways for cyanine -based NIR fluorescent probes, including IR-546, often involve OATP-dependent pathways, a characteristic shared by mitochondrial-targeting NIR probes (Xu et al., 2017; Wang et al., 2021). Given the partial mediation of IR-546 uptake by OATP transporters, which are commonly associated with mitochondrial-targeting probes. In addition, Next, we sought to confirm whether IR-546 specifically targets the mitochondria.

We incubated A375 and B16-F10 melanoma cells with both Mito-Tracker and Lyso-Tracker alongside IR-546, respectively to analyze the overlap of fluorescence signals and determine the intracellular targeting of IR-546 to mitochondria and lysosomes (Fig. 2G, S6 A). After 2 h of incubation, quantitative fluorescence analysis revealed that the green fluorescence from mitochondria (Mito-Tracker) overlapped more significantly with the red fluorescence of IR-546 compared to the lysosomes (Lyso-Tracker) (Figs. 2H, I, S6B, C). These results indicate that once internalized, IR-546, primarily targets the mitochondria in melanoma cells, confirming its mitochondrial specificity.

### IR-546 induces apoptosis of melanoma cells by affecting mitochondrial function

**Fig. 3:**
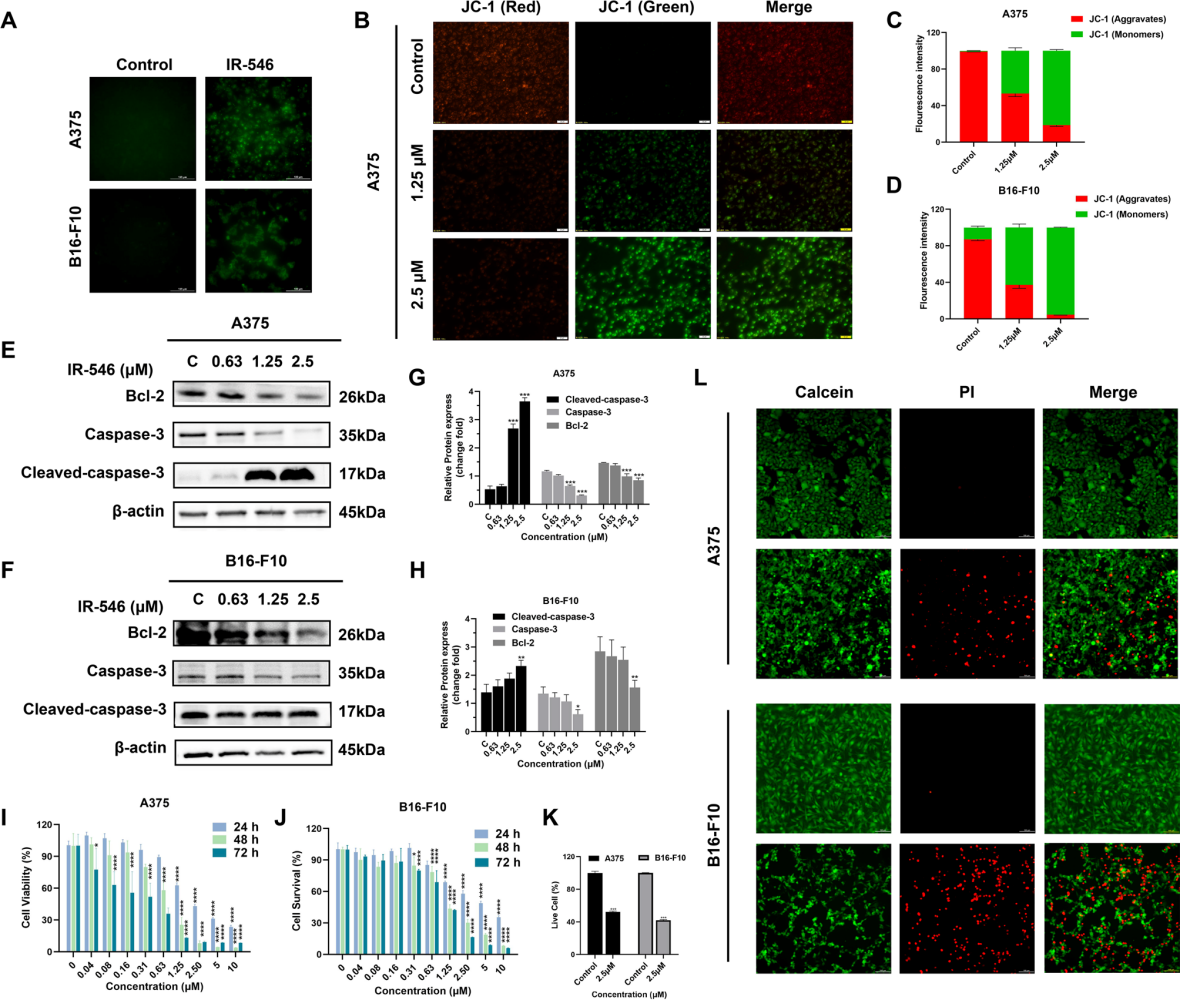
IR-546 induces apoptosis of melanoma cells by affecting mitochondrial function. **A** IR-546 induces ROS production in A375 and B16-F10 cells. **B** Detection of MMP in A376 melanoma cells treated with 1.25 µM and 2.5 µM IR-546, bar = 50 μm. **C** and **D** Ratio of JC-1 aggregates to JC-1 monomers in A375 and B16-F10 melanoma cells treated with 1.25 µM and 2.5 µM IR-546. **E** and **F** Apoptosis-related protein levels of Bcl-2, Caspase-3 and Cleaved-caspase-3 in A375 and B16-F10 melanoma cells treated with different concentrations of IR-546 for 24 h. β-actin was used as an internal reference. **G** and **H** Statistical analysis of protein expression levels of Bcl-2, Caspase-3, and Cleaved caspase-3 in figures **E** and **F**. **I** and **J** Cytotoxic effects of IR-546 to A375 and B16-F10 melanoma cells in a dose-dependent manner at 24 h, 48 h, and 72 h, respectively. **K** and **L** Indicate the dead/live fluorescent staining images of A375 and B16-F10 cells, scale bar = 100 μm. Each result was mean ± SD, *n* = 3. (****P < 0.0001, *** *P* < 0.001, ** *P* < 0.01, * *P* < 0.05, compared with control)

ROS can promote the intrinsic apoptosis pathway mediated by mitochondria (Idelchik et al., 2017). To assess whether IR-546 influences ROS production, intracellular ROS levels were measured in melanoma cells after a 2 h co-incubation with IR-546 (Fig. 3A). We found that IR-546 could induce intracellular ROS production in A375 and B16-F10 melanoma cells. Mitochondrial membrane potential (MMP, ΔΨm) disruption is a hallmark of mitochondrial dysfunction (Eirin et al., 2014). The decrease in MMP was evidenced by the induction of mitochondrial apoptosis (He et al., 2021). To investigate if IR-546 induces MMP collapse, A375 and B16-F10 melanoma cells were treated with 1.25 µM and 2.5 µM concentrations of IR-546, respectively. The results showed a decrease in red fluorescence intensity from JC-1 aggregates, coupled with an increase in green fluorescence from JC-1 monomers (Fig. 3B-D, S7) following IR-546 treatment. This change was concentration-dependent, indicating that IR-546 disrupts mitochondrial membrane potential, leading to MMP collapse.

Apoptosis is triggered by two main pathways, the intrinsic apoptotic pathway linked to mitochondrial oxidative stress and the extrinsic pathway, associated with cell death ligands (DO, 2023, Tuo et al., 2022). Activation of GSK3β phosphorylation can enhance the permeability of the mitochondrial outer membrane, triggering the intrinsic apoptosis pathway (Beurel and Jope, 2006, Grimes and Jope, 2001). The anti-apoptotic protein Bcl-2 is primarily located in the mitochondria and regulates outer membrane permeability, activating the Caspase cascade and leading to cell death (Peña-Blanco and García-Sáez 2018; Czabotar and Garcia-Saez, 2023). Caspase-3, a key protein in both intrinsic and extrinsic apoptotic pathways, is essential for executing apoptosis (Lossi et al., 2018, D’Amelio et al., 2010). As shown in Fig. 3E and F, IR-546 significantly inhibited the expression of the anti-apoptotic protein, Bcl-2, and increased the levels of Cleaved-caspase 3 in both A375 and B16-F10 melanoma cells in a concentration-dependent manner (Fig. 3G, H). These findings suggest that IR-546 induces apoptosis by targeting mitochondria, generating ROS, disrupting MMP, and activating the mitochondrial apoptotic pathway.

We used MTT assay to screen the cytotoxicity of IR-546 against A375, B16-F10, COS-1, MDA-MB-231, HeLa and A549 cells. We found that IR-546 had better inhibitory toxicity against melanoma at 72 h (Fig. S8). we further assessed the cytotoxicity of IR-546 in melanoma cells, an MTT assay was conducted. IR-546 was effectively taken up by melanoma cells and inhibited their proliferation in a concentration and time-dependent manner (Fig. 3I, J). Meanwhile, we observed that there was no aggregation induced quenching and good stability of IR-546 at the applied concentration (Fig. S9). To further evaluate its therapeutic potential, dead/live staining was performed. In the control group, only green fluorescence (live cells) was observed. In contrast, after treatment with 2.5 µM IR-546, both red (dead cells) and green fluorescence signals were detected in the experimental groups A375 and B16-F10 cells. The appearance of red fluorescence confirmed melanoma cell death post-treatment (Fig. 3L). Quantitative analysis showed that the percentage of live cells after IR-546 treatment group was 52% in A375 and 41% in B16-F10 cells (Fig. 3K). These results demonstrate that IR-546 has a promising therapeutic effect against melanoma.

### IR-546 inhibited the metastasis of melanoma cells

**Fig. 4:**
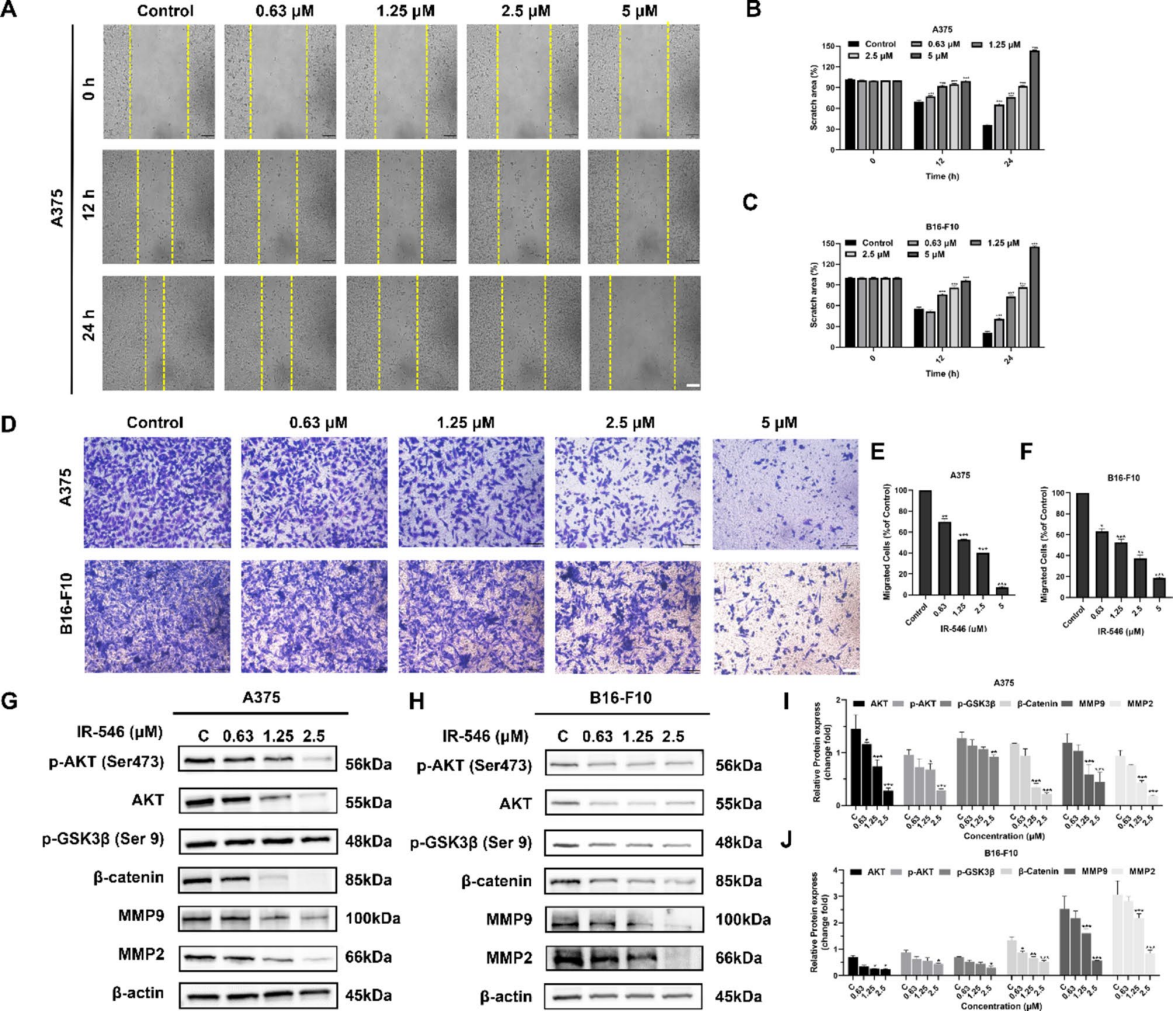
IR-546 inhibits the metastasis of A375 and B16-F10 melanoma cells. **A **In vitro wound healing assay the representation of the image, IR-546 can inhibit the migration and invasion of A375 melanoma cells, scale bar = 100 μm. **B** and **C** Quantitative analysis of the percentages of A375 and B16-F10 melanoma cell migration rate after being treated with IR-546 in different groups, scale bar = 50 μm. **D**, **E** and **F** Representative photographs of transwell migration assay of A375 and B16-F10 melanoma cells and results of quantitative analysis, scale bar = 50 μm. **G** and **H** Metastasis-related protein levels of AKT, p-AKT, p-GSK3β, β-catenin, MMP9, and MMP2 in A375 and B16-F10 melanoma cells treated with different concentrations of IR-546 for 24 h. β-actin was used as an internal reference. **I** and **J** A375 and B16-F10 melanoma cells were treated with different concentrations of IR-546 for 24 h, and the protein levels of AKT, p-AKT, p-GSK3β, β-catenin, MMP9, and MMP2 were statistically analyzed. Each result was mean ± SD, *n* = 3. (****P < 0.0001, *** *P* < 0.001, ** *P* < 0.01, * *P* < 0.05, compared with control)

Based on the previous experimental results, we selected IR-546 concentrations below 5 µM for follow-up studies in A375 and B16-F10 melanoma cells. In the wound healing assay, IR-546 significantly inhibited the migration of melanoma cells (Figs. 4A-C, S10). At 5 µM, the scratch width increased, likely due to IR-546’s cytotoxic effects on A375 and B16-F10 cells. we also selected A2058 melanoma cells to examine the effect of IR-546 on cell migration ability of A2058. Firstly, the results showed that IR-546 could rapidly enter into A2058 cells (Fig. S11 A). Subsequently, we observed that IR-546 significantly inhibited the migration of A2058 melanoma cells. Notably, at concentrations of 2.5 μM and 5 μM, A2058 cells exhibited complete mortality by 12 h, further demonstrating the cytotoxic effect of IR-546. (Fig. S11B). Similarly, in the transwell assay, IR-546 markedly reduced the number of migrating melanoma cells (Fig. 4D-F). These results demonstrate that IR-546 decreases the horizontal and vertical migration capacity of melanoma cells.

The serine/threonine kinase AKT primarily regulates the phosphorylation of GSK3β (Tsai et al., 2022, Cross et al., 1995). As a mature substrate of AKT (Marwarha et al., 2022), GSK3β, upon phosphorylation at ser9, can further phosphorylate β-catenin, leading to its degradation by the proteasome complex, thereby affecting downstream transcription (Dai et al., 2024). Therefore, we investigated the role of the AKT/GSK3β pathway to understand the molecular mechanism of IR-546’s effects.

To explore these mechanisms, we analyzed proteins downstream of AKT and GSK3β involved in metastasis. IR-546 treatment downregulated the expression of AKT, p-AKT, p-GSK3β, and β-catenin (Fig. 4G, H). Matrix metalloproteinases, MMP9 and MMP2, key downstream molecules regulated by β-catenin (Han et al., 2023), play critical roles in tumor metastasis and invasion (Deng et al., 2022). IR-546 suppressed the expression of MMP2 and MMP9 in a concentration-dependent manner (Fig. 4I, K). This suggests that IR-546 effectively suppresses key proteins involved in melanoma cell migration and metastasis.

In conclusion, IR-546 inhibits melanoma cell proliferation by activating the mitochondrial apoptosis pathway through suppression of the AKT/GSK3β signaling pathway. Additionally, IR-546 downregulates β-catenin and its downstream effectors, MMP9 and MMP2, leading to reduced metastasis of A375 and B16-F10 melanoma cells. Therefore, IR-546 exerts antitumor and anti-metastatic effects by targeting the AKT/GSK3β pathway.

### IR-546 inhibits the growth of melanoma without obvious side effects in vivo

**Fig. 5:**
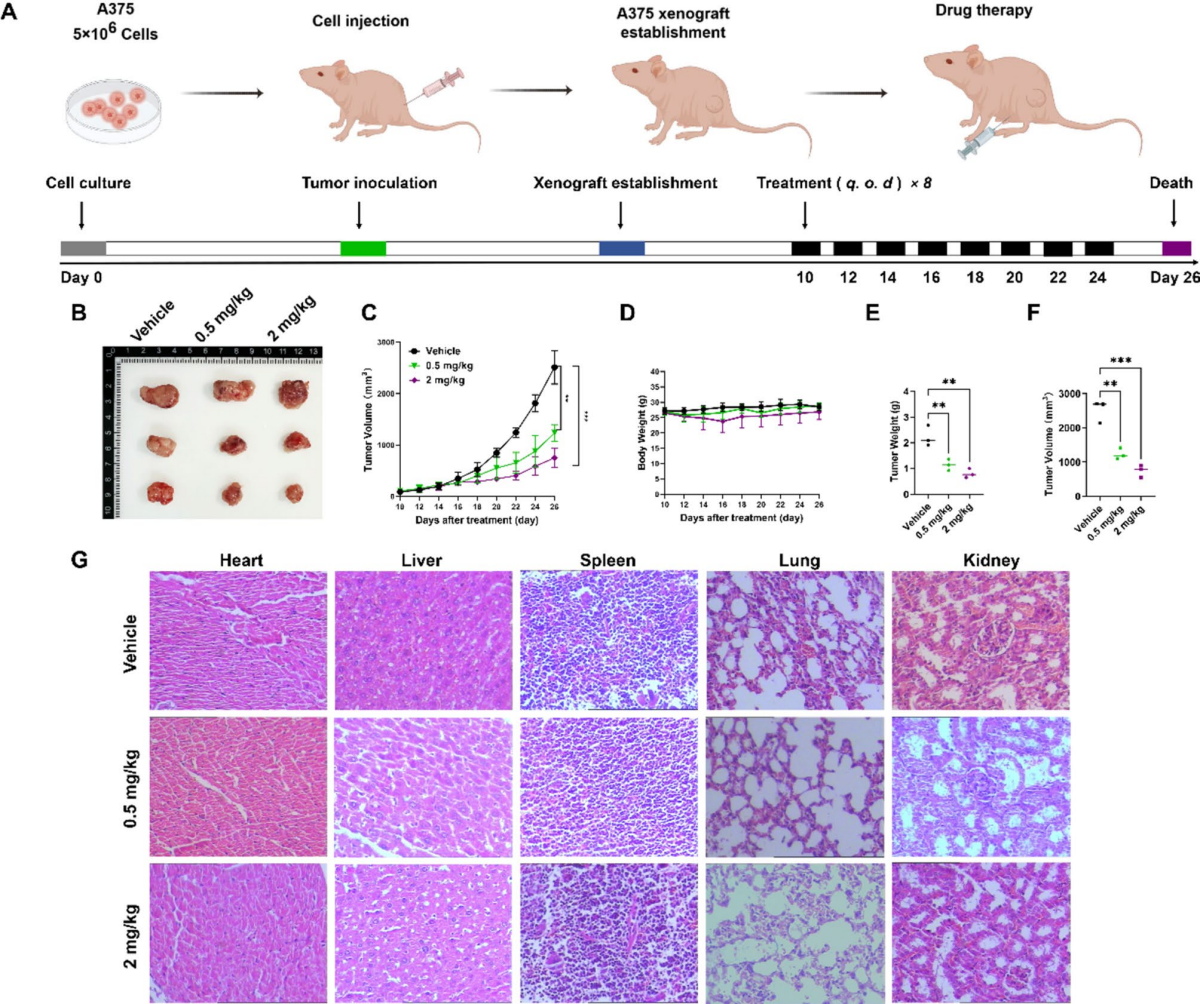
IR-546 inhibits the growth of melanoma and has good biosafety in vivo. **A** Schematic for experimental design. **B** Imaging of the dissected mouse tumor. **C** Growth curves for A375 human malignant melanoma cells in mice treated with different concentrations of IR-546. **D** Weight of mice in the process of treatment. Tumor weight (**E**) and tumor volume (**F**) on the last day. **G** H&E staining of heart, liver, spleen, lung, and kidney of BALB/c mice model (A375). Each result was mean ± SD, *n* = 3. (****P < 0.0001, *** *P* < 0.001, ** *P* < 0.01, * *P* < 0.05, compared with control)

To further assess the impact of IR-546 on melanoma growth we conducted in vivo experiments to evaluate its antitumor efficacy. As shown in Fig. 5A, we divided the tumor-bearing mice into three groups and treated them every other day for eight treatment cycles. Tumor growth was significantly inhibited in both low and high-concentration IR-546 groups compared to the control group, with effects becoming evident around day 7 and progressively intensifying over time (Fig. 5B, C). Interestingly, while the high-concentration group exhibited transient weight loss on day 7 and then recovered, which may be related to the potential toxicity of the drug. The low-concentration group did not show any significant weight changes during the treatment period. This suggests that a lower dose of IR-546 may be more cost-effective and safer for inhibiting tumor growth in vivo (Fig. 5D). At the end of the experiment, tumor volume and weight were substantially reduced in the low and high-concentration treatment groups, compared to the control treatment group (Fig. 5E, F), indicating that IR-546 has potent anti-tumor activity in vivo. Histological analysis through H&E staining revealed no significant morphological abnormalities in the organs of mice such as heart, liver, spleen, lung, and kidney treated with IR-546, confirming that IR-546 had good biosafety in vivo (Fig. 5G).

### Anti-tumor mechanisms of IR-546 in vivo

**Fig. 6:**
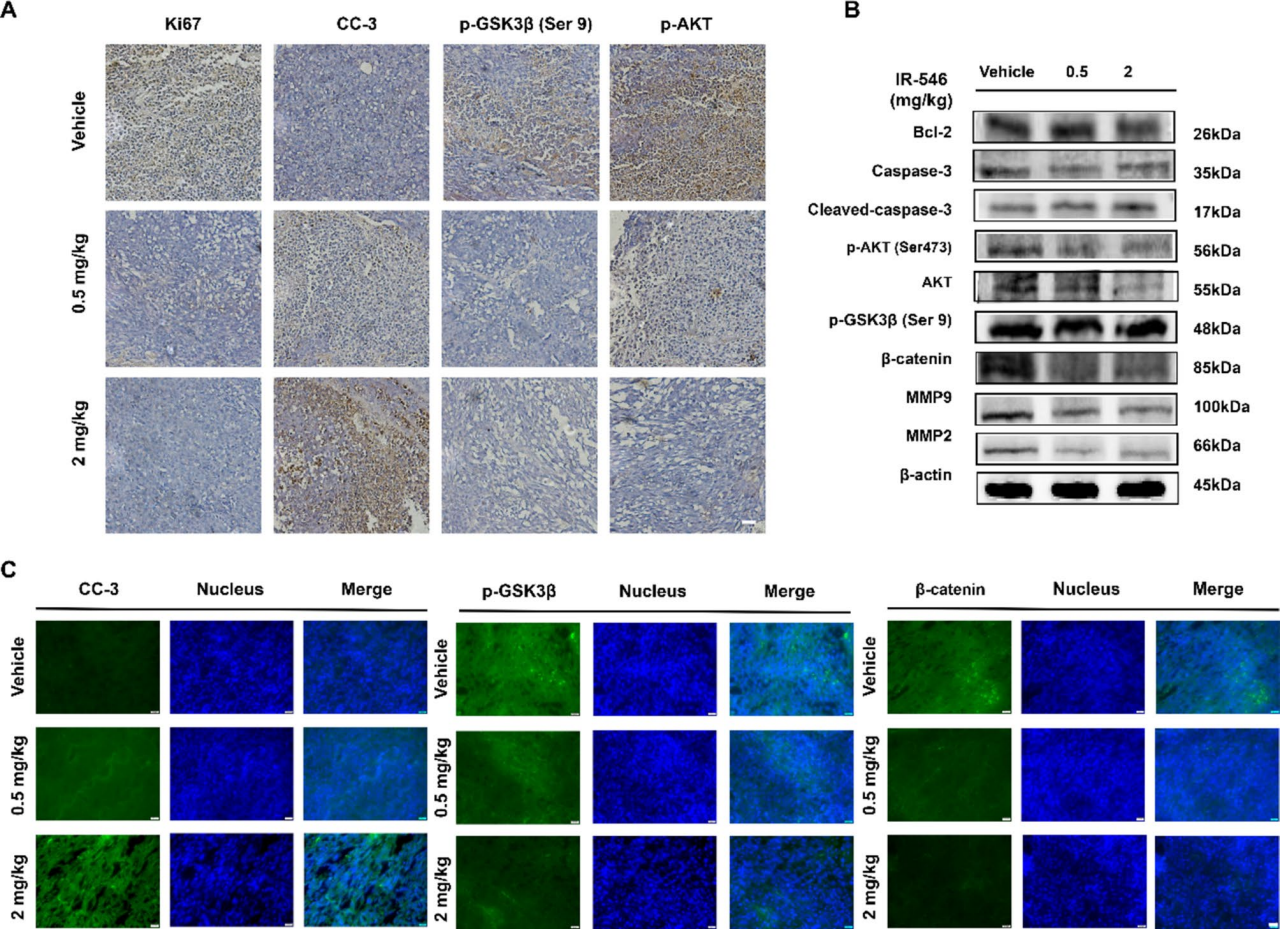
Anti-tumor mechanisms of IR-546 in vivo. **A** Representative images of the tumor sections were used for Ki67, CC-3, p-GSK-3β (Ser9) and p-AKT staining, Scale = 50 μm. **B** The expression levels of AKT, p-AKT, p-GSK-3β (Ser9), β-catenin, MMP9, and MMP2 in tumor of BALB/c mice model (A375) were detected by western blot. **C** CC-3, p-GSK-3β (Ser9), and β-catenin were detected by immunofluorescence, Scale = 50 μm. Each result was mean ± SD, *n* = 3. (****P < 0.0001, *** *P* < 0.001, ** *P* < 0.01, * *P* < 0.05, compared with control)

Cleaved caspase-3 (CC-3), Caspase-3 cleaved at an aspartic acid residue to produce p12 and p17 subunits, which are activated forms of the latter and are responsible for morphological and biochemical changes in apoptosis (Silva et al., 2022). Immunohistochemical analysis revealed that IR-546 inhibited the expression of Ki67, CC-3, p-GSK-3β (Ser9), and p-AKT in tumors from A375 tumor-bearing mice in a concentration-dependent manner (Fig. 6A). These findings are consistent with our earlier in vitro results. Additionally, western blot analysis of tumor tissues demonstrated that IR-546 effectively suppressed tumor growth via the AKT/GSK-3β/β-catenin pathway (Fig. 6B). Immunofluorescence analysis further confirmed that IR-546 reduced the expression of key proteins CC-3, p-GSK-3β (Ser9), β-catenin, and MMP2 (Fig. 6C, S12). These data suggest that IR-546 inhibits human malignant melanoma A375 growth in vivo by targeting the AKT/GSK-3β/β-catenin signaling pathway.

## Discussions

Malignant melanoma (MM) can be widely found in various tissues of the body. They have a high degree of malignancy and strong invasiveness. In the field of diagnosis and treatment of melanoma, significant progress has been made in the application of fluorescent probes in diagnosis, surgical resection and sentinel lymph node localization and resection (Wang et al., 2021). Biomarker-activated fluorescent probes, especially near-infrared fluorescent probes, provide a powerful means for the diagnosis and treatment of melanoma (von Kiedrowski et al., 2020). In this study, we synthesized a novel NIR fluorescent probe named IR-546 based on the hemicyanine structure through design, chemical modification and activity screening. First, we explored the optical properties of IR-546 in different solvent systems. The results indicated that the fluorescence wavelengths of IR-546 in the five solutions of 10% FBS, 0.9% NaCl, PBS, DMSO and MeOH were within the range of the “tissue window” (Shell et al., 2015). It has near-infrared fluorescence characteristics (Fig. 2B). Meanwhile, the emission wavelength of IR-546 at 650 nm is conducive to reducing the absorption of light by hemoglobin and melanin and is beneficial for improving the signal-to-noise ratio and tumor targeting (Vickerman et al., 2021). Among them, the fluorescence intensity of IR-546 in PBS and 0.9% NaCl solutions was weak and lower than that in DMSO and MeOH solutions. This phenomenon may be related to the low solubility of IR-546 in the first two solvents. It should be noted that there are differences in the spectroscopic characteristics of the NIR fluorescent probes in the current study in 10% FBS solution compared to the NIR fluorescent probes in our previous study (Wang et al., 2023a; Sun et al., 2022). The fluorescence intensity of IR-546 in 10% FBS solution was significantly enhanced, which was better than that in DMSO and MeOH solutions. This phenomenon may be attributed to the better binding of IR-546 to albumin in fetal bovine serum to form nanoparticles, resulting in fluorescence enhancement. The use of 10% FBS as a solvent also has potential application in in vivo bioimaging diagnosis and therapy, which also provides a new idea for material selection, optimization and design to improve the in vivo fluorescence imaging ability of IR-546.

Mitochondria, which are regarded as ideal anti-tumor drug targets (Weinberg and Chandel, 2015, Guo et al., 2021). Compared with normal cells, hyperpolarized tumor cell membranes and mitochondrial membrane potential allow lipotropic cations and the coupling of the selective accumulation in tumor cells of mitochondria (Huang et al., 2021). Cyanine dyes have similar structural characteristics to some lipophilic polyamines (Herlan et al., 2021, Nichugovskiy et al., 2022) and mitochondrial-targeting peptides (Nam et al., 2018, Schultz and Nevler, 2022). Therefore, cyanine fluorescent probes can be selectively enriched in tumor cells based on mitochondrial membrane potential driving, reflecting the advantages of the integration of tumor imaging and targeted therapy. In addition, increasing the asymmetry of cyanine dye molecules can improve their signal-to-noise ratio (Tian et al., 2023). Recent reports have shown that increasing the asymmetry of Cy7 can enhance tumor targeting to a certain extent (Zeng et al., 2025). Therefore, the novel asymmetric near-infrared fluorescent probe IR-546 with lipophilic cations that we synthesized is beneficial for targeting mitochondria. Co-localization analysis of IR-546 with mitochondria (Mito-Tracker) and lysosomes (Lyso-Tracker) was performed in A375 cells, respectively. The Manders coefficients M1 (IR-546) and M2 (Mito-Tracker) were 0.725 and 0.924, and M1 (IR-546) and M2 (Lyso-Tracker) were 0.594 and 0.712. Similarly, in B16-F10 cells, the Manders coefficients M1 (IR-546) and M2 (Mito-Tracker) were 0.712 and 0.987, and M1 (IR-546) and M2 (Lyso-Tracker) were 0.882 and 0.945. A higher Manders coefficient for IR-546/Mito-Tracker indicates that IR-546 colocalizes more strongly with Mito-Tracker than with Lyso-Tracker (Garg et al., 2012). These values confirm IR-546 could target to the mitochondria of melanoma cells.

Next, we investigated the cell-killing effect of IR-546 on melanoma cellsin vitro and in vivo. MTT assay showed that the IC_50_ of IR-546 for A375 melanoma cells at 24 h, 48 h, and 72 h was 2.4, 0.7, and 0.2 µM, respectively, and for B16-F10 melanoma cells was 4.3, 1.7, and 1.0 µM, respectively. Excellent antitumor activity can be observed. In the A375 BALB/c mice xenograft model, compared with the control group, the low concentration and high concentration of IR-546 had a significant killing effect on melanoma cells, and the tumor inhibition rates were 50% and 70%, respectively. The low concentration group had no significant change in body weight during the whole treatment process, showing a safer and more efficient anti-tumor ability in vivo. Immunohistochemical analysis of the tumor tissue also showed decreased Ki67 expression, indicating decreased cell proliferation, and increased CC-3 expression, indicating increased apoptotic cells, which were consistent with previous in vitro results. Therefore, IR-546 has a good tumor-killing effect in vivo and in vitro.

The curative effect of melanoma difference is the result of melanoma cells high transfer ability. If the metastasis of melanoma can be effectively inhibited, the therapeutic effect of melanoma will be significantly enhanced (Liu et al., 2022). We also explored the potential anti-tumor metastatic mechanism of IR-546 in melanoma. In recent years, AKT/GSK3β/β-catenin signaling axis plays an important role in inhibiting the proliferation, invasion, migration, improving immunogenicity and reducing drug resistance of melanoma (Abbas et al., 2024; Jere et al., 2019). Therefore, we speculate that this signal axis has great research potential in the diagnosis and treatment of melanoma. AKT inhibits the activity of GSK3β by phosphorylating its Ser9 terminus, and GSK3β inhibits cell migration by further phosphorylating β-catenin and inhibiting its entry into the nucleus and affecting the expression of downstream target genes (Dai et al., 2024). Wang et al. demonstrated that SLFN5 inhibits melanoma cell migration and invasion by inhibiting MT1-MMP expression through the AKT/GSK3β/β-catenin signaling axis (Wan et al., 2019). Furthermore, this signal axis is also related to mitochondrial energy metabolism and the induction of apoptosis (Yang et al., 2017). Xu et al. reported that mitochondrial driven apoptosis in the retina was reduced by upregulation of the AKT/GSK3β/β-catenin signaling axis (Xu et al., 2022). In this study, We observed that IR-546 could significantly inhibit the horizontal and vertical migration of melanoma cells in a concentration-dependent manner through wound healing and transwell experiments. The increased migration width and significantly reduced cell number in the 5 µM treatment group also indicated that IR-546 could simultaneously induce the apoptosis of melanoma cells. Thus, IR-546 inhibited melanoma metastasis by inhibiting AKT/GSK3β/β-catenin in vitro.

In this study, the novel NIR fluorescent probe IR-546 synthesized by us can target the mitochondria of melanoma. By targeting mitochondria, IR-546 can directly disrupt the energy metabolism center of melanoma cells, and show efficient and precise killing effect on melanoma in vivo and in vitro. Despite its excellent performance in tumor killing, moderate fluorescence image and potential biotoxicity of IR-546 are the two major issues. Optimizing the fluorescence imaging ability can better improve the application range of IR-546 in clinical diagnosis, while reducing the biological toxicity may affect its safety and efficacy in the treatment process. Therefore, how to optimize the fluorescence imaging performance and reduce the biotoxicity while maintaining the high tumor killing ability of IR-546 has become two major issues that need to be paid attention to. Therefore, further design improvements and material optimization of IR-546 to enhance its fluorescence imaging capabilities and antitumor effects warrant continued investigation.

## Conclusions

This study successfully developed a novel mitochondrial-targeting NIR fluorescent probe, IR-546. It exhibits near-infrared fluorescence, which aligns with the “tissue optical window” characteristics. This makes the IR-546 a promising tool for tumor imaging. The probe is preferentially internalized by melanoma cells through energy-dependent metabolic pathways. Once inside, it targets mitochondria, inducing ROS production. disrupting MMP, and triggering the mitochondrial apoptosis pathway to induce tumor cell death. IR-546, demonstrated potent antitumor in vivo, with no observable side effects. Furthermore, the probe showed significant anti-metastatic effects in melanoma cells. Mechanistically, IR-546 activated GSK-3β Ser9 phosphorylation, which suppressed β-catenin and its downstream proteins in vitro and in vivo, thereby inhibiting melanoma growth through suppression of the AKT/GSK-3β/β-catenin pathway.

## Supplementary Information

Supplementary Material 1.

Supplementary Material 2.

## Data Availability

No datasets were generated or analysed during the current study.
