# Supplementary material for: Novel NIR fluorescent probe IR-546 inhibits melanoma through the AKT/GSK3β/β-catenin pathway
Source: Mol Med. 2025 Jun 10;31:226. doi: 10.1186/s10020-025-01289-0 (PMC12150457; doi:10.1186/s10020-025-01289-0)
Supplement: Supplementary file 1 — Supplementary Material 1. [file 10020_2025_1289_MOESM1_ESM.docx]

**Supporting information**

**Novel NIR fluorescent probe IR-546 inhibits melanoma through the AKT/GSK3β/β-catenin pathway**

Hongye Liao^a,1^, Tong Xia ^a,1^, Ziyuan Zeng ^a,1^, Xun Yang^a^, Simei Yang ^b^, Xia Xiong^a^, Yuanmin He^a^, Na Hao ^c*^, Li Liu ^a*^

a Department of Dermatology, The Affiliated Hospital, Southwest Medical University, Luzhou, 646000, China.

b School of Public Health, Southwest Medical University, Luzhou, 646000, China

c Green Pharmaceutical Technology Key Laboratory of Luzhou, Central Nervous System Drug Key Laboratory of Sichuan Province, Department of Medicinal Chemistry, School of Pharmacy, Southwest Medical University, Luzhou 646000, China.

* Corresponding author.

E-mail address:

[haona@swmu.edu.cn](mailto:haona@swmu.edu.cn) (N. Hao)

[liuli@swmu.edu.cn](mailto:liuli@swmu.edu.cn) (L. Liu),

**^1^** These authors contributed equally to this work.


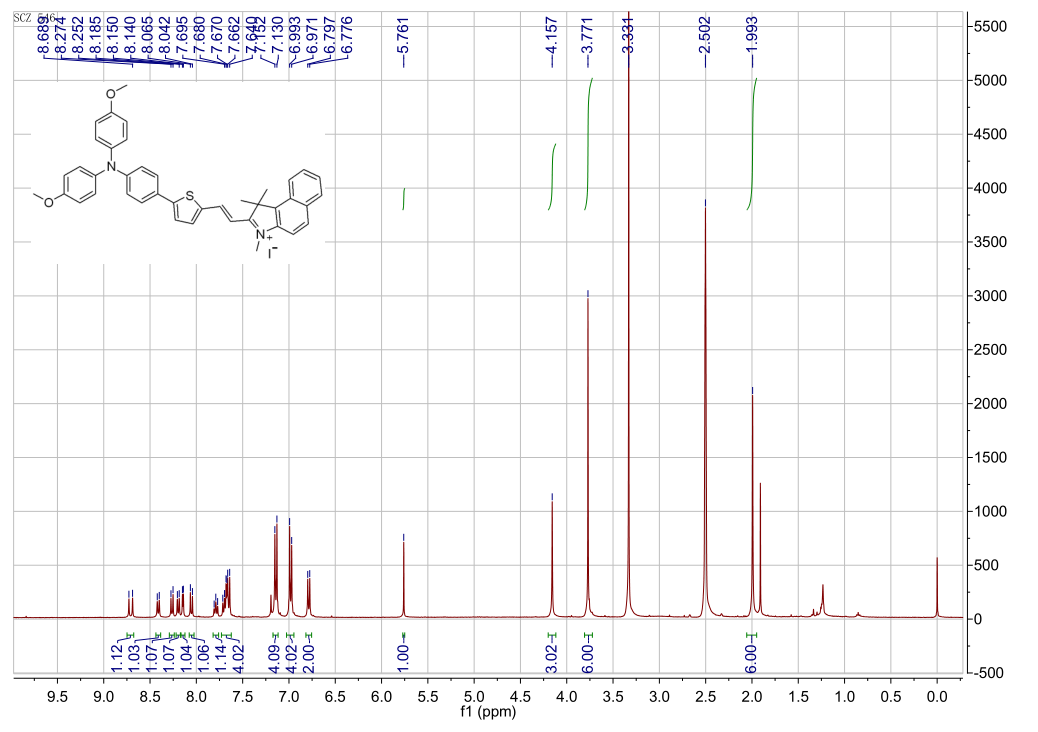
**Figure S1.** The ^1^H NMR spectrum of IR-546.


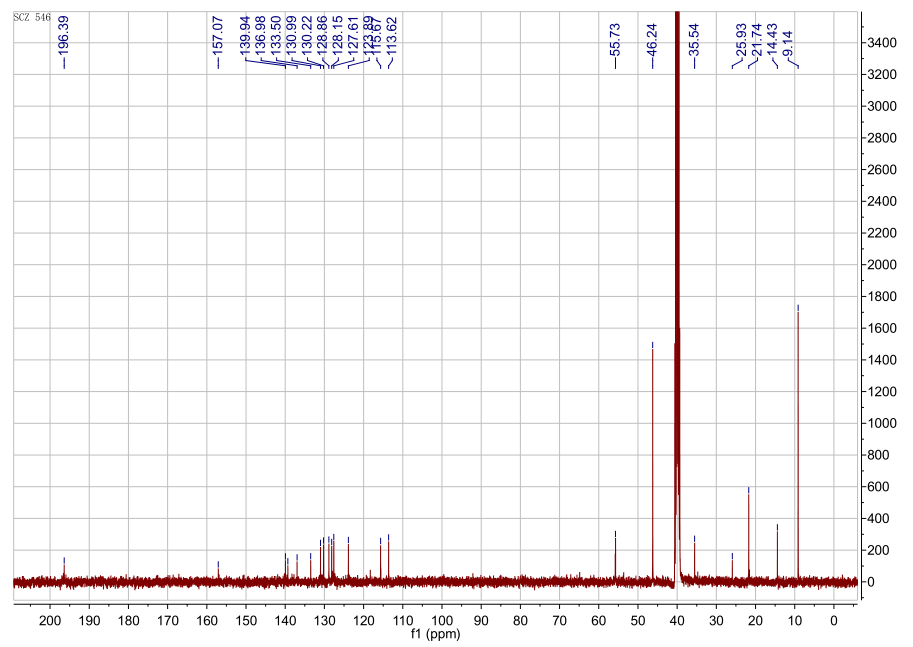


**Figure S2.** The ^13^C NMR spectrum of IR-546.


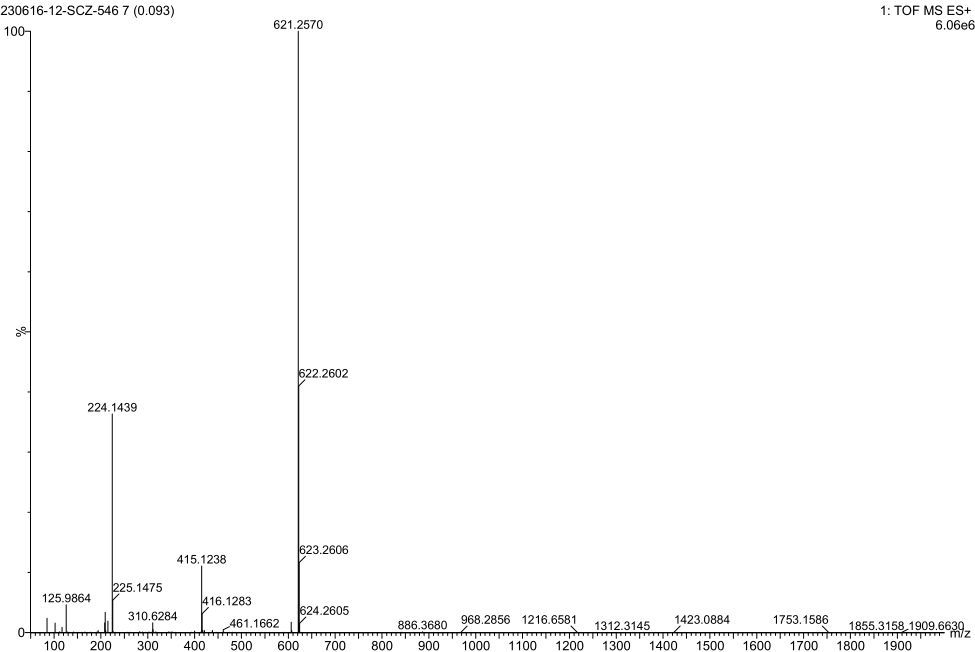
**Figure S3.** The HMRS spectrum of IR-546.


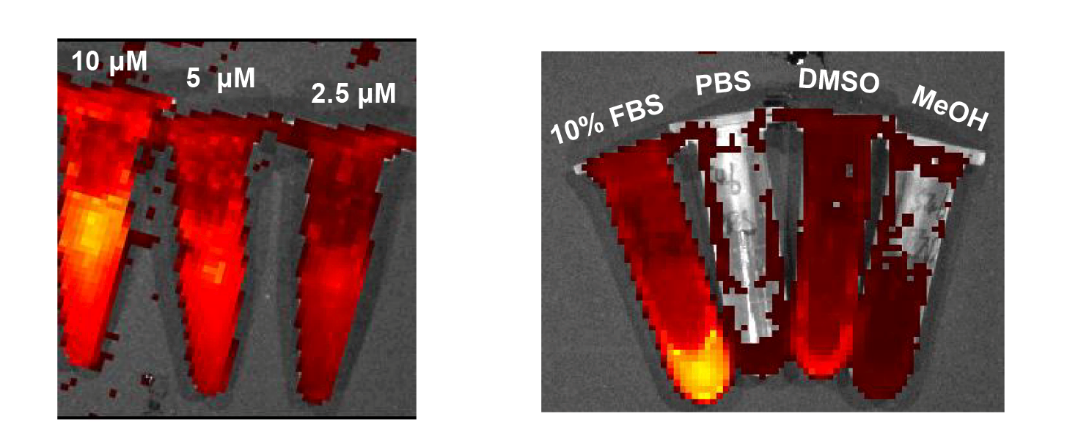


**Figure S4.** Fluorescence imaging of 10 μM IR-546 in 10% FBS, PBS, DMSO and MeOH solutions.

**
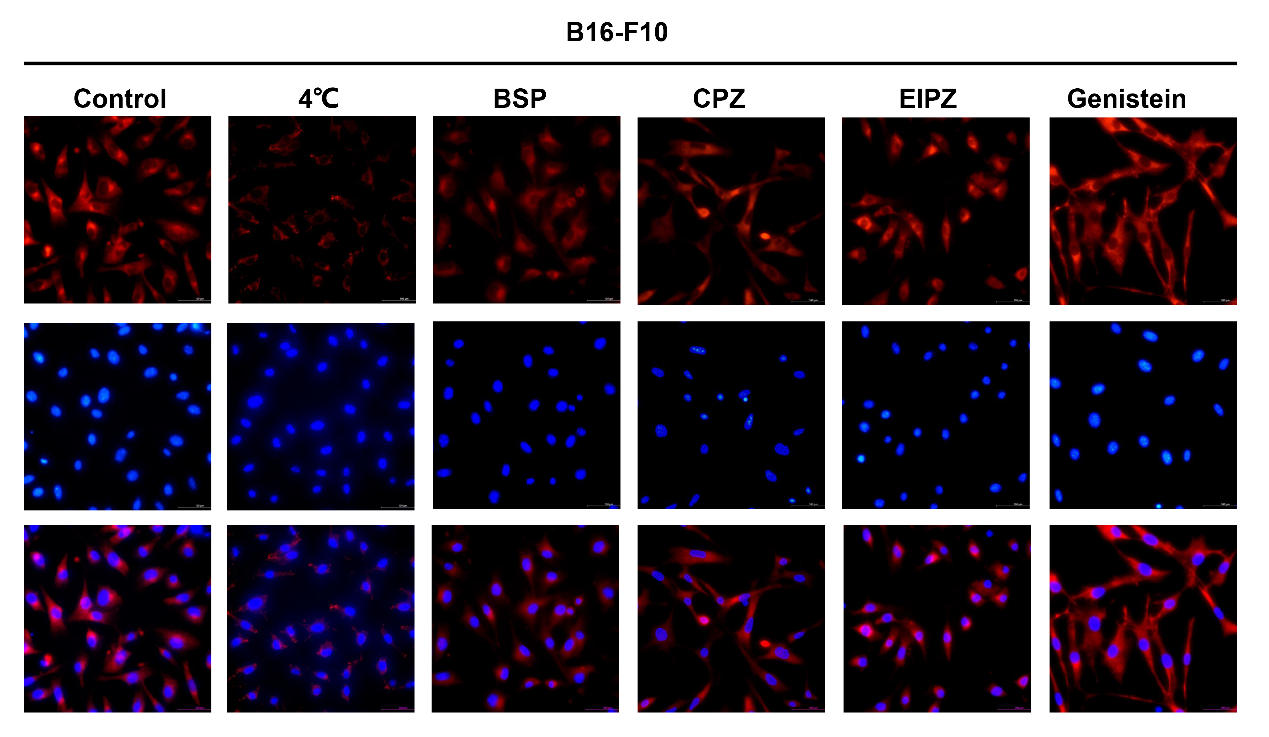
**

**Figure S5.** Fluorescence images of B16-F10 melanoma cells after incubation with IR-546 as well as different endocytic inhibitors, scale bar = 100 μm.


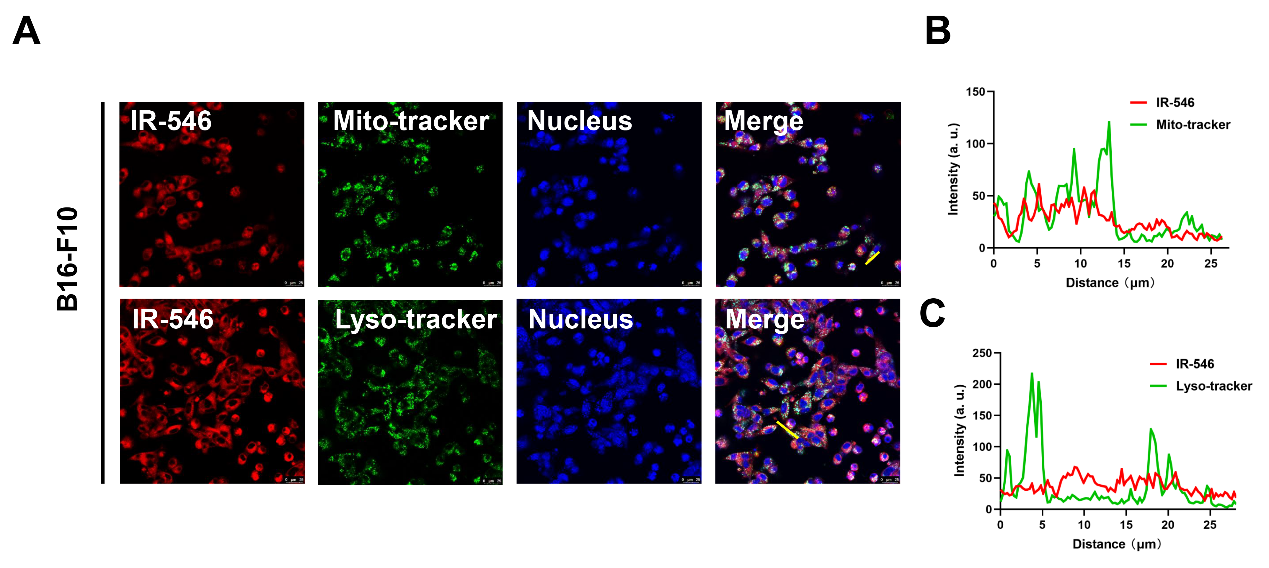


**Figure S6.** (A) Co-localization fluorescence imaging of IR-546 with Mito-Tracker and Lyso-Tracker in B16-F10 melanoma cells, respectively, bar = 25μm. (B) and (C) Show the colocalization analysis of IR-546 in mitochondria and Lysosome in B16-F10 melanoma cells, respectively.


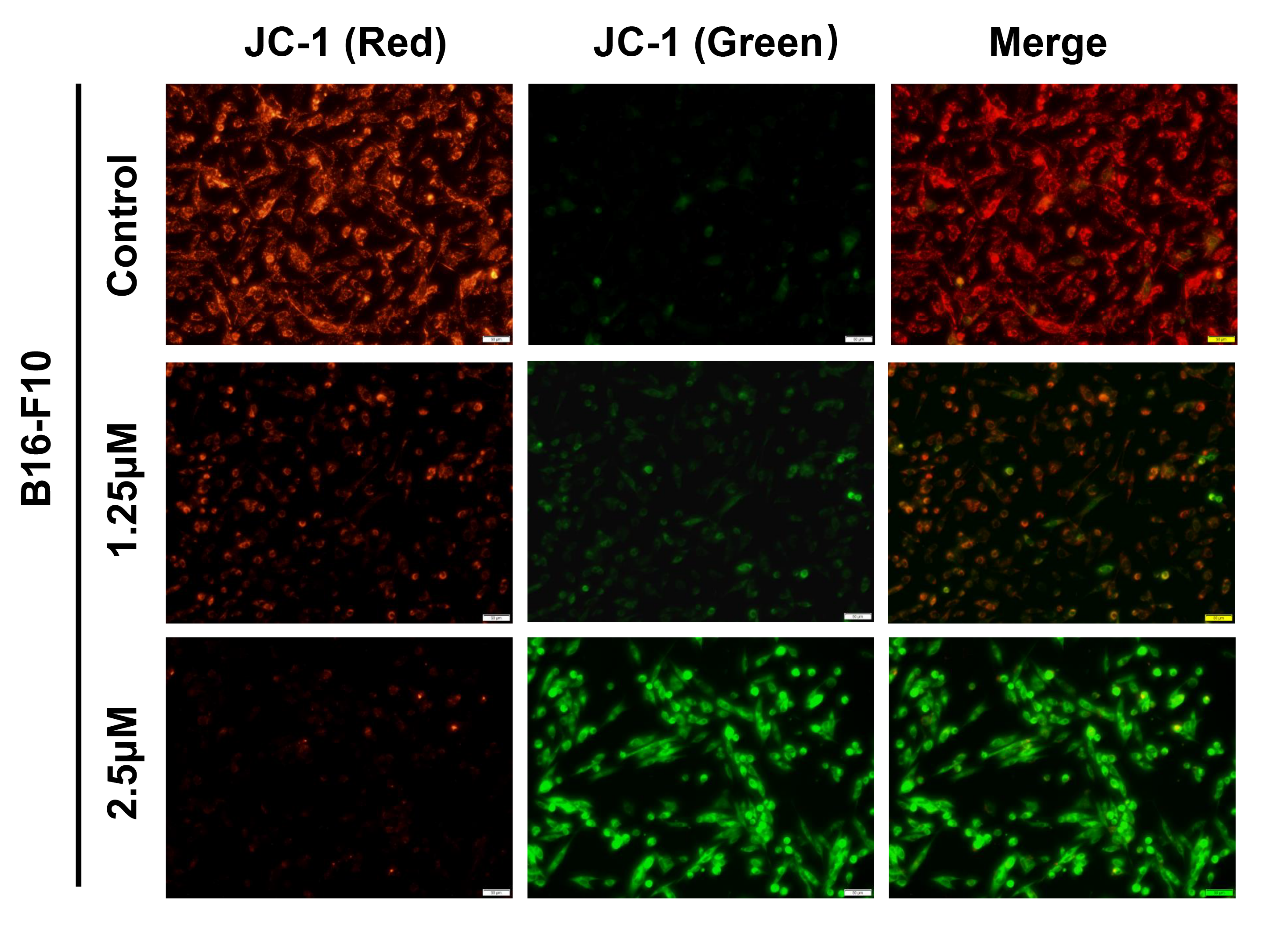


**Figure S7.** Fluorescence imaging of JC-1 aggregates and JC-1 monomers in B16-F10 melanoma cells treated with 1.25 μM and 2.5 μM IR-546, bar = 50 μm.


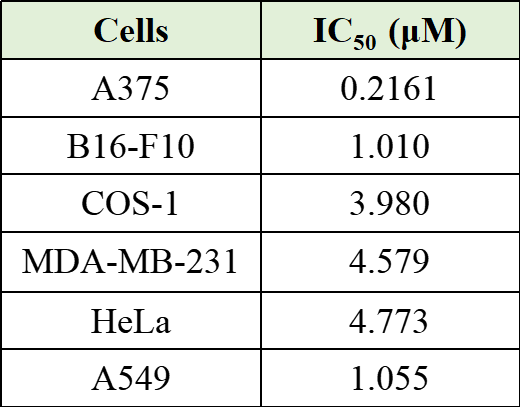

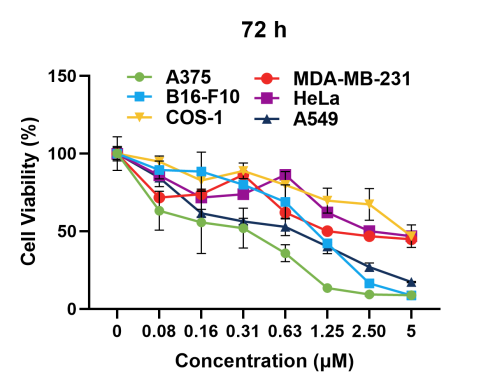


**Figure S8**. Cytotoxic effects of IR-546 to A375, B16-F10, COS-1, MDA-MB-231,HeLa and 549cells in a dose-dependent manner at 72 h.

**
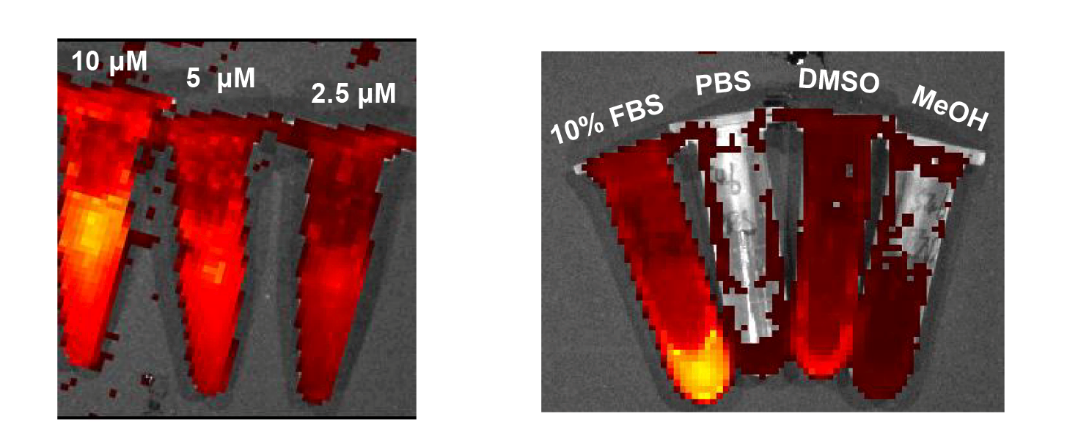
**

**Figure S9.** Fluorescence stability of IR-546 at treatment concentration.


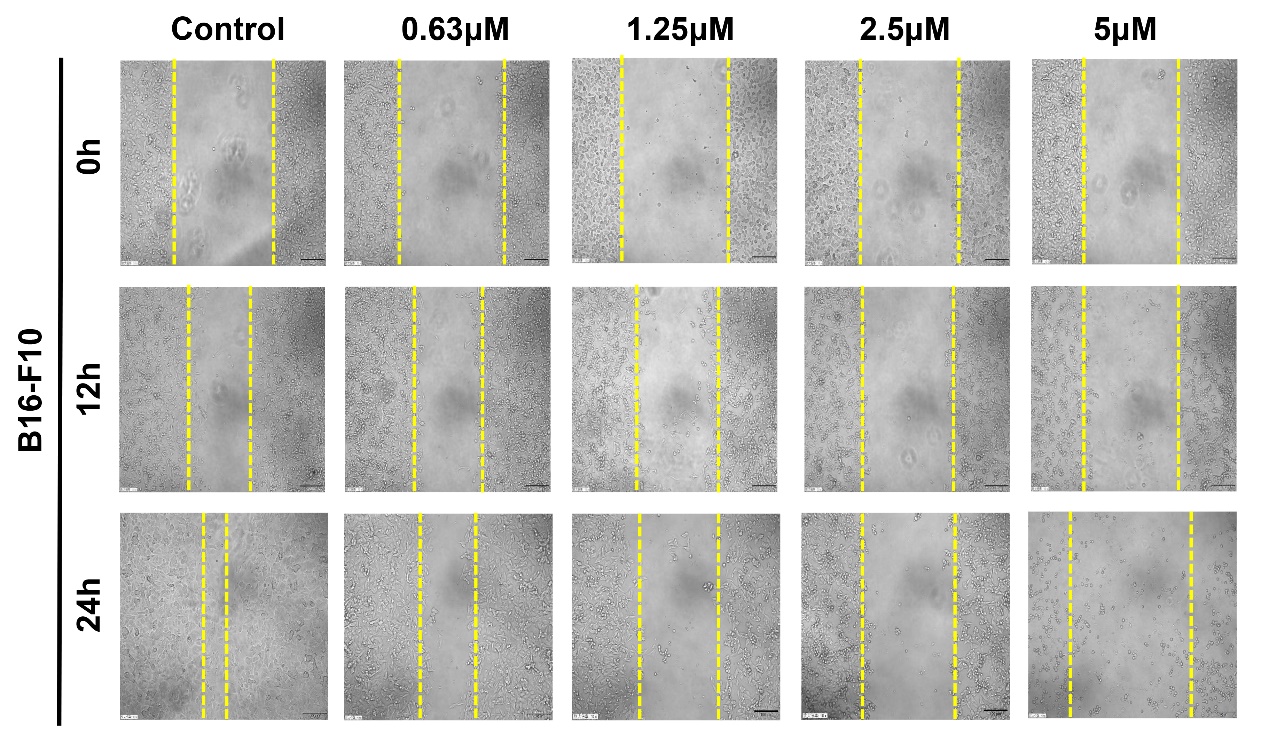


**Figure S10.** *In vitro* wound healing assay the representation of the image, IR-546 can inhibit the migration and invasion of B16-F10 melanoma cells, scale bar = 100 μm..


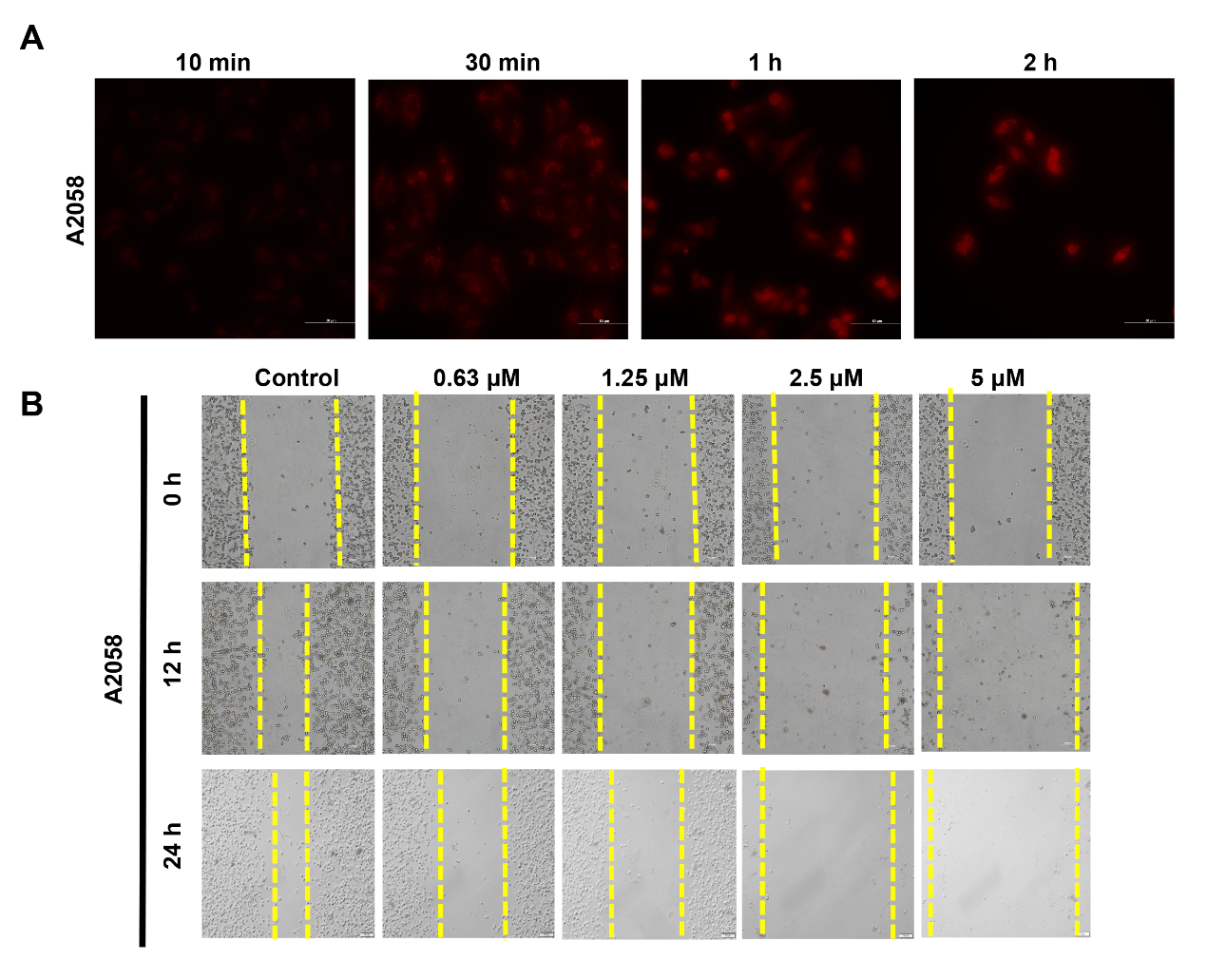
**Figure S11.** (A) Uptake of IR-546 by A2058 melanoma cells at different time points (10 min, 30 min, 1 h, 2 h), Scale bars, 25 μm. (B). The images of wound healing at 0 h, 12 h, and 24 h in A2058 cells after being treated with different concentrations of IR-546. Scale bars, 100 μm.


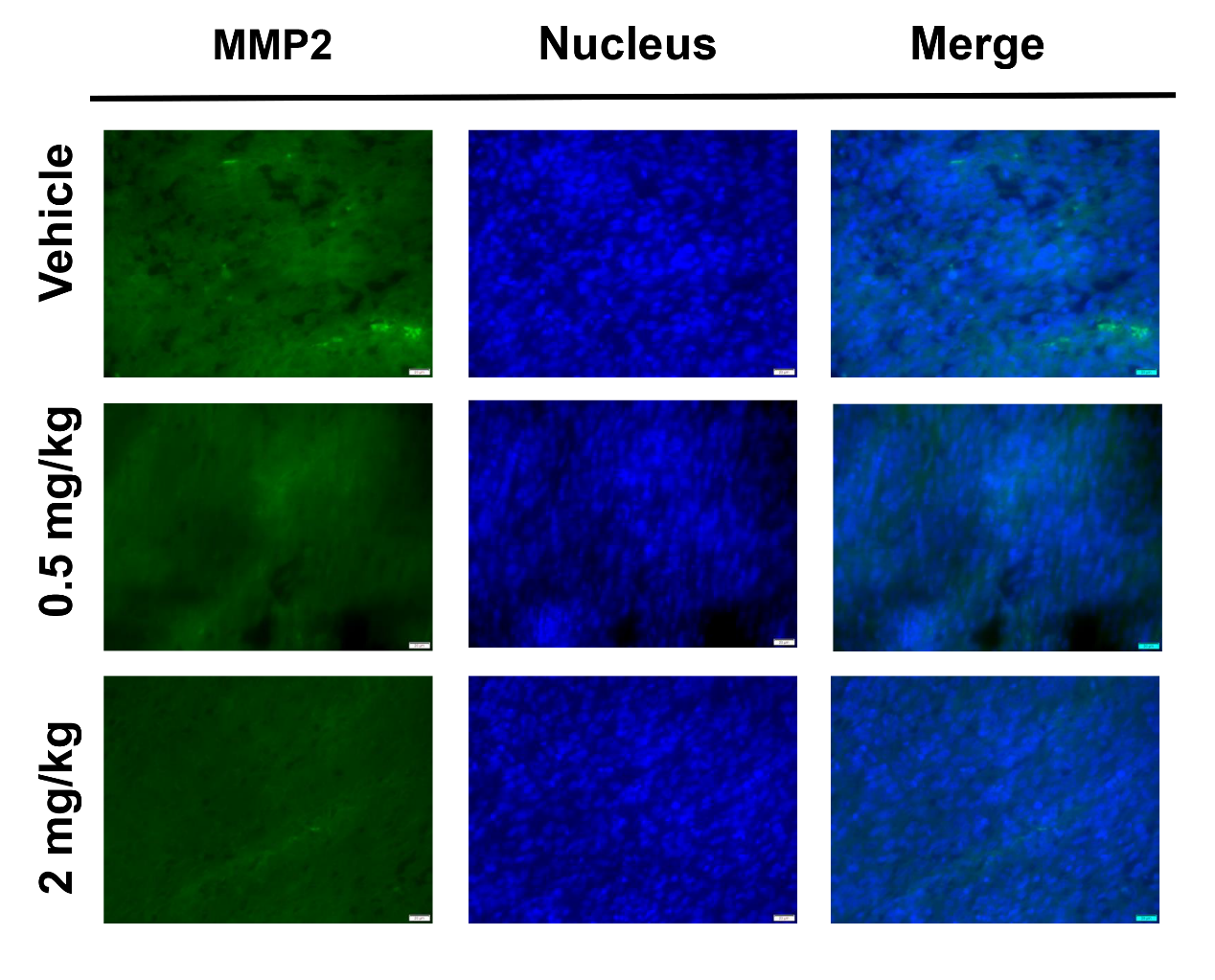


**Figure S12.** MMP2 was detected by immunofluorescence, Scale=50 μm.
